# No Evidence for a Trade-Off between Reproductive Investment and Immunity in a Rodent

**DOI:** 10.1371/journal.pone.0037182

**Published:** 2012-05-23

**Authors:** Yan-Chao Xu, Deng-Bao Yang, De-Hua Wang

**Affiliations:** 1 State Key Laboratory of Integrated Management of Pest Insects and Rodents, Institute of Zoology, Chinese Academy of Sciences, Beijing, China; 2 Graduate School of the Chinese Academy of Sciences, Beijing, China; National Cancer Institute, United States of America

## Abstract

Life history theory assumes there are trade-offs between competing functions such as reproduction and immunity. Although well studied in birds, studies of the trade-offs between reproduction and immunity in small mammals are scarce. Here we examined whether reduced immunity is a consequence of reproductive effort in lactating Brandt's voles (*Lasiopodomys brandtii*). Specifically, we tested the effects of lactation on immune function (Experiment I). The results showed that food intake and resting metabolic rate (RMR) were higher in lactating voles (6≤ litter size ≤8) than that in non-reproductive voles. Contrary to our expectation, lactating voles also had higher levels of serum total Immunoglobulin G (IgG) and anti-keyhole limpet hemocyanin (KLH) IgG and no change in phytohemagglutinin (PHA) response and anti-KLH Immunoglobulin M (IgM) compared with non-reproductive voles, suggesting improved rather than reduced immune function. To further test the effect of differences in reproductive investment on immunity, we compared the responses between natural large (n≥8) and small litter size (n≤6) (Experiment II) and manipulated large (11–13) and small litter size (2–3) (Experiment III). During peak lactation, acquired immunity (PHA response, anti-KLH IgG and anti-KLH IgM) was not significantly different between voles raising large or small litters in both experiments, despite the measured difference in reproductive investment (greater litter size, litter mass, RMR and food intake in the voles raising larger litters). Total IgG was higher in voles with natural large litter size than those with natural small litter size, but decreased in the enlarged litter size group compared with control and reduced group. Our results showed that immune function is not suppressed to compensate the high energy demands during lactation in Brandt's voles and contrasting the situation in birds, is unlikely to be an important aspect mediating the trade-off between reproduction and survival.

## Introduction

Reproduction and self-maintenance are important for fitness and both require substantial energy investment [1], [2], [3], [4], [5], [6]. Because animals are frequently constrained by intrinsic physiological limitations that govern their capacity to expend energy, they must consequently maintain an optimal allocation of energy between competing physiological functions (e.g. growth, reproduction and immunity) [7], [8].

In small mammals, the costs of reproduction involve higher energy and nutrient demands and energy expenditure [5]. The energy demands of mammalian reproduction increase throughout lactation; particularly late lactation is the energetically critical period of the breeding cycle [9]. The greater expenditure during lactation is related to the mass of nursing young and to the cost of their locomotion and temperature regulation, as well as to the cost of growth [10], [11]. Organ remodeling which involves growth of the alimentary tract and other associated metabolic organs (including heart, liver, lung and kidney) and body fat utilization are necessary to achieve the high demands of lactation in many small rodents [10], [12]. A number of hormones may play an important role in the energy intake and expenditure during lactation. Leptin, secreted by white adipose tissue, is known to be involved in the regulation of food intake during lactation [13], [14]. In addition, prolactin is required for the ongoing maintenance of milk secretion [15] and the regulation of hyperphagia and metabolic process during lactation [16]. These two hormones may also play an important signal driving counterbalance between reproduction and immune function [4]. Elevated corticosterone release may reflect the stress of high energy demand [17], which may suppress immunity [18]. The high cost of lactation requires that energy intake must increase, or that the allocation of energy to other functions reduces [19]. However, sustained energy intake during late lactation might be limited intrinsically by aspects of an animal's physiology [9], [11], [12], [20]; other physiological functions would be consequently down-regulated.

Life-history theory predicts that current reproductive effort gives rise to a fitness cost, which may be observed as reduced survival or future reproduction [21]. To survive, animals must be able to generate immune responses to resist potentially life-threatening diseases. However, mounting an immune response requires substantial energy [1], [3], [4], [22]. Many studies in lots of species have found support for trade-offs between reproduction and immunity, with immunity being suppressed during energetically reproductive periods [23], [24], [25], [26], [27], although not all studies have showed a suppresive effect of reproductive investment on immune function [28]. In a variety of species, when investment in reproduction increases, there is a concomitant increase in host susceptibility to parasites [29], [30].

The precise reasons for immunosuppression during reproductive period are unclear, but one proposed mechanism considered reproductive effort as the main reason to suppress immune function [31]. Many studies have documented that experimentally increased reproductive effort adversely affected immune function in birds [2], [24], [25], [30], [31], [32], [33], [34]. Few studies have focused on the effect of reproductive effort during lactation on the ability to mount immune responses in small mammals [35], [36].

We conducted three experiments to test the hypothesis whether the reproductive effort of lactating Brandt's voles (*Lasiopodomys brandtii*) negatively affects the immune function. We predicted that i) the immune function would be suppressed in lactating voles, and ii) larger reproductive effort (larger litters) would be associated with greater immunosuppression.

## Results

### Experiment I

#### Body mass, food intake and resting metabolic rate (RMR)

**Figure 1:**
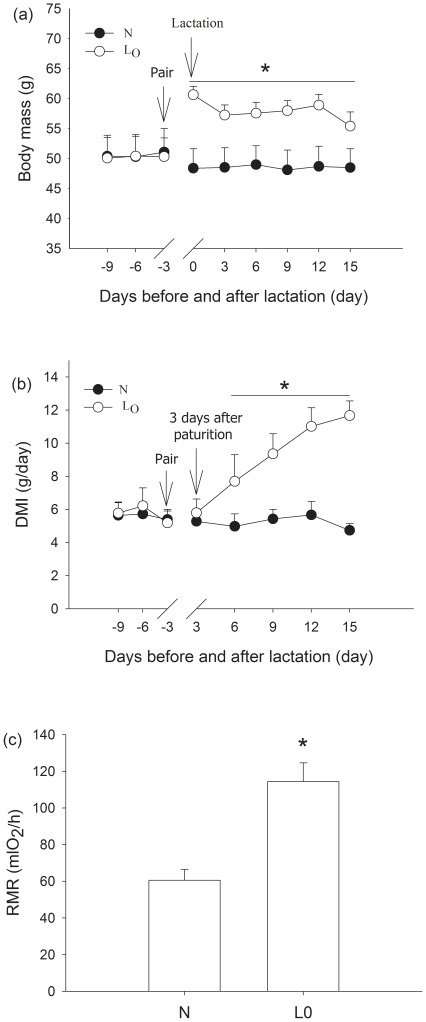
Changes of body mass (a) and dry matter intake (DMI) (b) over time. RMR (c) on day 13 of lactation in lactating voles. Values are means ± s.e.m. N represents non-lactating group, and L_0_ represents lactating group. Significant difference between groups is indicated by an asterisk if P<0.05.

Before pairing, there was no significant difference in body mass between the non-lactating and lactating voles (t = 0.140, df = 10, *P* = 0.892; Figure 1a). The lactating voles had higher body mass than non-lactating voles during 15 days of lactation (group effect, *F_1,11_* = 6.348, *P* = 0.028; day effect, *F_5,55_* = 2.222, *P* = 0.065; interaction group×day, *F_5,55_* = 2.401, *P* = 0.049; Fig. 1a).

Before pairing, there was no significant difference in dry matter intake between the non-lactating and lactating voles (t = 0.211, df = 10, *P* = 0.837). The lactating voles had significantly higher dry matter intake than non-lactating voles from day 6 to day 15 during lactation (group effect, *F_1,10_* = 20.956, *P*<0.001; day effect, *F_4,40_* = 7.631, *P*<0.001; interaction group×day, *F_4, 40_* = 8.211, *P*<0.001; Fig. 1b), and dry matter intake was increased by about 100% compared with non-lactating voles on day15.

RMR in lactating voles was also significantly increased by about 100% compared with non-lactating voles (*F_1, 10_* = 13.147, *P* = 0.05; Fig. 1c) on day 13.

#### Serum hormones

Serum leptin concentrations were significantly decreased in lactating voles compared to non-lactating voles (t = 2.440, df = 10, *P* = 0.035; table 1). Lactating voles had higher serum prolactin concentrations than non-lactating voles (t = −2.976, df = 11, *P* = 0.013; table 1). There was no significant difference in serum corticosterone concentrations between lactating voles and non-lactating voles (t = −2.146, df = 10, *P* = 0.057; table 1).

**Table 1:** Effect of lactation on serum hormones in female Brandt's voles.

Parameters	N	L_0_	T	df	P
Leptin (ng/ml)	4.434±0.617^a^	2.717±0.217^b^	2.440	10	P<0.05
Prolactin (ng/ml)	134.611±9.873^a^	176.673±16.305^b^	−2.976	11	P<0.05
Corticosterone (nmol/l)	2.984±0.174	3.801±0.339	−2.146	10	Ns

Values are means ± s.e.m. significant differences are indicated by different superscripts in each row if P<0.05, determined by independent-samples T test.

#### Body composition and organ mass

Thymus mass was reduced in lactating voles compared to non-lactating voles (*F_1, 10_* = 8.438, P = 0.016; Table S1), whereas spleen mass had no significant difference (*F_1, 10_* = 0.01, *P* = 0.921). Body fat mass (*F_1, 10_* = 6.335, *P* = 0.031) and body fat content (*F_1, 10_* = 6.115, *P* = 0.043) were decreased in lactating voles compared with non-lactating voles. Liver mass (*F_1, 10_* = 13.856, *P* = 0.004), gastrointestinal tract with or without content were increased in lactating voles compared with non-lactating voles except caecum mass (*F_1, 10_* = 3.551, *P* = 0.089). There were no significant differences in heart (*F_1, 10_* = 0.019, *P* = 0.893), lungs (*F_1, 10_* = 0.048, *P* = 0.831), kidneys (*F_1, 10_* = 2.219, *P* = 0.167) between the two groups.

#### Innate immunity, PHA response and humoral immunity

**Figure 2.The:**
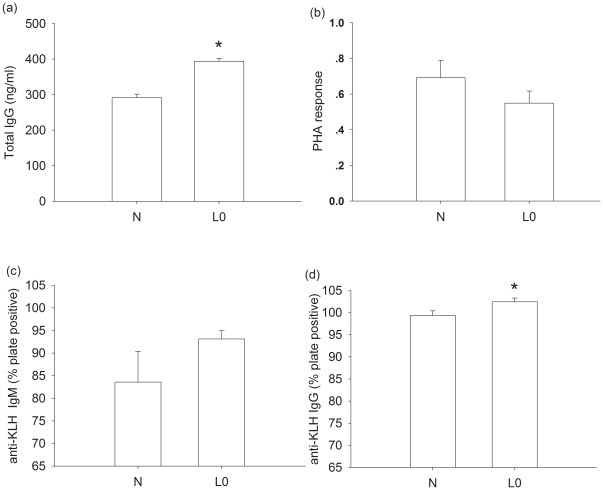
The effects of lactation on serum total IgG (a), PHA response (b), serum anti-KLH IgM (c) and anti-KLH IgG (d) in N and L_0_ group. Values are means ± s.e.m. Significant difference between groups is indicated by an asterisk if P<0.05.

Serum total Immunoglobulin G (IgG) concentration was significantly increased in lactating females (t = −8.328, df = 11, *P*<0.001; Fig. 2a) compared with non-lactating voles. No significant difference in PHA response was found between non-lactating and lactating voles (t = 1.174, df = 11, *P* = 0.265; Fig. 2b). There was no significant difference in anti-keyhole limpet hemocyanin (KLH) Immunoglobulin M (IgM) concentrations between non-lactating and lactating voles (t = −1.341, df = 12, *P* = 0.205; Fig. 2c). Lactating voles had significantly higher anti-KLH Immunoglobulin G (IgG) concentrations (t = −2.311, df = 12, *P* = 0.039; Fig. 2d) compared with non-lactating voles.

### Experiment II

#### Litter size and litter mass

**Figure 3:**
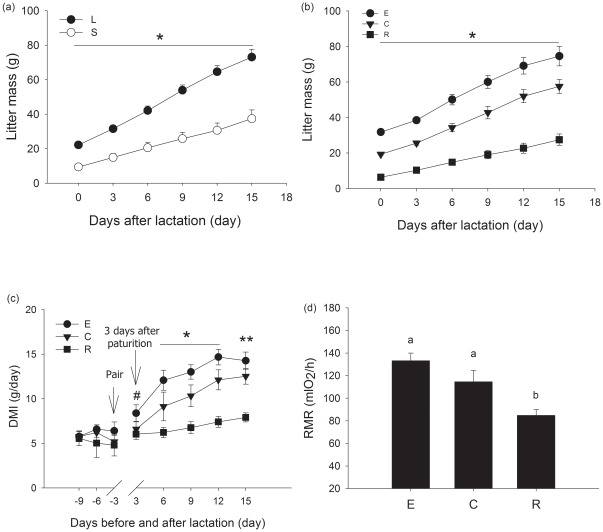
Changes of litter mass (a) during lactation. L represents large litter size group, and S represents small litter size group. Changes of litter mass (b) after manipulation during lactation. E represents enlarged litter size group, C represents non-manipulated litter size group, and R represents reduced litter size group. Changes of dry matter intake (DMI) (c) over time and RMR (d) on day 13 of lactation. Values are means ± s.e.m. Significant differences in litter mass between L and S group or litter mass and food intake between E, C and R are indicated by an asterisk if P<0.05. A pound sign indicates significant differences in DMI between E and R group, and a double asterisk indicates significant differences between E and R group, and between C and R group if P<0.05, whereas significant differences in RMR between E, C and R are indicated by different letters if P<0.05.

At the end of lactation, the mean numbers of offspring in large and small groups were 9.0±0.0 and 3.3±0.5, respectively (Fig. 3a). Females in the large litter size group (L) had a significantly higher total litter mass than those in the small litter size group (S) (group effect, *F*_1,15_ = 31.772, *P*<0.001; day effect, *F*_5,75_ = 178.721, *P*<0.001; interaction group×day, *F*_5,75_ = 17.449, *P*<0.001; Fig. 3a). The mean pup mass in large and small groups were 2.471±0.043 g and 2.909±0.103 g on day 0 of lactation and 8.122±0.507 g and 12.066±0.785 g on day 15 of lactation, respectively. The mean pup mass in the large litter size group was smaller than that in the small litter size group (group effect, *F_1,15_* = 16.675, *P* = 0.001; day effect, *F_5,75_* = 232.576, *P*<0.001; interaction group×day, *F_5,75_* = 11.239, *P*<0.001).

#### Maternal body mass

Before pairing, no difference in body mass existed between the Large and small group (t = 2.059, df = 15, *P* = 0.059). During lactation, change of body mass in large group was not significantly different from that of small group (group effect, *F_1, 15_* = 4.416, *P* = 0.053; day effect, *F_5, 75_* = 16.941, *P*<0.001; interaction group×day, *F_5, 75_* = 2.598, *P* = 0.032; Fig. S2).

#### Serum hormones

Serum leptin concentration had no significant difference in large litter size voles, compared to small litter size voles (t = 0.532, df = 15, *P* = 0.519; Table S2). An increased trend, but no significant difference in serum prolactin was found in large litter size voles compared to small litter size voles (t = 0.556, df = 15, *P* = 0.587; Table S2). There was no significant difference in serum corticosterone concentrations between large litter size voles and small litter size voles (t = 1.459, df = 10, *P* = 0.175; Table S2).

#### Body composition and organ mass

Thymus (*F_1, 14_* = 0.068, *P* = 0.798) and spleen mass (*F_1, 14_* = 0.058, *P* = 0.813) did not differ between large litter size voles and small litter size voles (Table S2). There were no significant differences in body fat mass (*F_1, 14_* = 1.632, *P* = 0.222), body fat content (*F_1, 14_* = 1.172, *P* = 0.297), heart (*F_1, 14_* = 0.407, *P* = 0.534), liver (*F_1, 14_* = 2.250, *P* = 0.156), lungs (*F_1, 14_* = 0.411, *P* = 0.532), kidneys (*F_1, 14_* = 3.806, *P* = 0.071) and gastrointestinal tract with or without content between the two groups except stomach (*F_1, 14_* = 7.476, *P* = 0.016) and intestine with content mass (*F_1, 14_* = 9.757, *P* = 0.007).

#### Innate immunity, PHA response and humoral immunity

**Figure 4:**
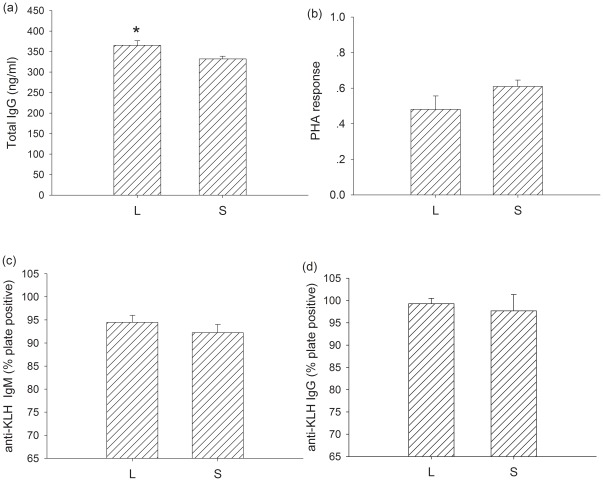
The effect of litter size on serum total IgG (a), PHA response (b), serum anti-KLH IgM (c) and serum anti-KLH IgG (d) in L and S group. Values are means ± s.e.m. Significant difference between groups is indicated by an asterisk if P<0.05.

Total IgG concentration was greater in large litter size females than small litter size females (t = 4.418, df = 15, *P*<0.001; Fig. 4a).There was no significant difference in the PHA response between the large litter size and small litter size individuals (t = −1.705, df = 15, *P* = 0.109; Fig. 4b). KLH-IgG (t = 0.278, df = 18, *P* = 0.784; Fig. 4d) and KLH-IgM (t = 0.218, df = 18, *P* = 0.830; Fig. 4c) concentrations were also not significantly different between large litter size and small litter size individuals.

### Experiment III

#### Litter size and litter mass

The mean numbers of offspring in enlarged, control and reduced groups were 11.7±0.2, 7.5±0.2 and 2.4±0.3 at the end of lactation (Fig. 3b). Total litter mass differed significantly among manipulation groups (group effect, *F_2,18_* = 61.373, *P*<0.001; day effect, *F_5,90_* = 190.617, *P*<0.001; interaction group×day, *F_10,90_* = 9.180, *P*<0.001; Fig. 3b). Specifically, the enlarged group had a significantly higher total litter mass than both the control and reduced groups (*P*<0.001), and the reduced group had a significantly lower total litter mass than the control group (*P*<0.001).

#### Maternal body mass, food intake and RMR

Before pairing, no difference in body mass existed among enlarged, control and reduced groups (*F_2, 18_* = 1.136, *P* = 0.343). During lactation, there was no significant difference among these three groups (group effect, *F_2, 18_* = 2.487, *P* = 0.111; day effect, *F_5, 90_* = 9.744, *P*<0.001; interaction group×day, *F_10, 90_* = 2.25, *P* = 0.022; Fig. S3). Further analysis showed that body mass in reduced group decreased significantly during this period (*F_5, 35_* = 10.660, *P*<0.001), but not in enlarged group (*F_5, 30_* = 2.251, *P* = 0.075) and control group (*F_5, 25_* = 2.145, *P* = 0.093).

Before mating, there was no significant difference in dry matter intake in enlarged, control and reduced groups (group effect, *F_2,18_* = 1.222, *P* = 0.318; day effect, *F_2,36_* = 0.835, *P* = 0.442; interaction group×day, *F_4,36_* = 1.083, *P* = 0.379; Fig. 3c). Significant differences were found among enlarged, control and reduced groups during lactation (group effect, *F_2,18_* = 17.716, *P*<0.001; day effect, *F_4,72_* = 41.777, *P*<0.001; interaction group×day, *F_8,72_* = 4.885, *P*<0.001). From day 3 to day 15 during lactation, food intake of all voles significantly increased (*P*<0.05). The voles with enlarged litters had significant higher dry matter intake than those of control voles (*P* = 0.030) except day 15 and the voles with reduced litters throughout lactation (*P*<0.001), and dry matter intake of the voles with reduced litters was significantly lower than control voles (*P* = 0.005).

Significant difference in RMR was found among enlarged, control and reduced groups (*F_2,15_* = 10.217, *P* = 0.002; Fig. 3d). Specifically, RMR of the voles raising reduced litters was significant lower than that of control voles (*P* = 0.015), but there was no significant difference between voles with enlarged litters and control voles (*P* = 0.104).

#### Serum hormones

There were no significant differences in serum leptin concentrations in enlarged voles, compared to control and reduced voles (*F_2, 18_* = 0.321, *P* = 0.730; Table S3). An increased trend, but no significant difference in serum prolactin was found between reduced group and enlarged group (*F_2, 18_* = 1.001, *P* = 0.387; Table S3). Serum corticosterone concentrations had no significant difference among these three groups (*F_2, 18_* = 0.843, *P* = 0.447; Table S3).

#### Body composition and organ mass

Thymus (*F_2, 17_* = 0.433, *P* = 0.656) and spleen mass (*F_2, 17_* = 0.368, *P* = 0.698) did not differ among enlarged litter size voles, control voles and reduced litter size voles (Table S3). The differences were not significant for body fat mass (*F_2, 17_* = 0.012, *P* = 0.988) and body fat content (*F*_2, 17_ = 0.007, *P* = 0.993). There were no significant differences in heart (*F_2, 17_* = 0.805, *P* = 0.463), liver (*F_2, 17_* = 1.337, *P* = 0.289), lungs (*F_2, 17_* = 0.618, *P* = 0.551), kidney (*F_2, 17_* = 2.017, *P* = 0.164), gastrointestinal tract with or without content among these three groups except stomach with content (*F_2, 17_* = 3.683, P = 0.047) and stomach (*F_2, 17_* = 5.676, P = 0.013).

#### Innate immunity, PHA response and humoral immunity

**Figure 5:**
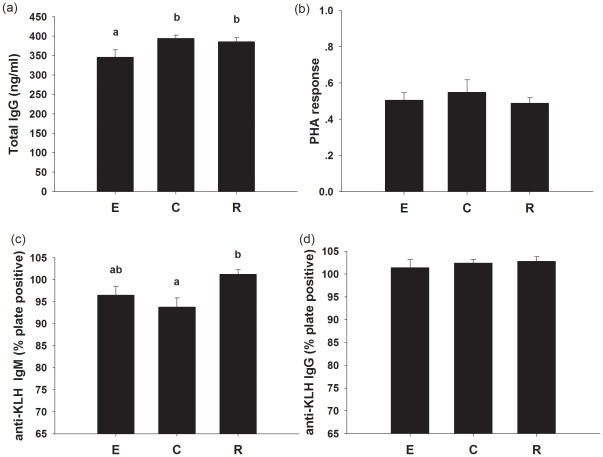
The effect of litter size manipulation on serum total IgG (a), PHA response (b), serum anti-KLH IgM(c) and serum anti-KLH IgG (d) in E, C and R group. Values are means ± s.e.m. Significant difference among groups is indicated by an asterisk if P<0.05.

Voles with enlarged litters had lower total IgG than voles that had their litters reduced and control voles (*F_2,18_* = 3.731, *P* = 0.042; Fig. 5a). PHA response was not significantly different among enlarged, control and reduced voles (*F_2,18_* = 0.452, *P* = 0.643; Fig. 5b). KLH-IgM of voles raising enlarged litters was not significantly different to that of control voles, however, voles with reduced litters had significantly higher anti-KLH IgM than controls (*F_2,17_* = 4.640, *P* = 0.025; Fig. 5c). KLH-IgG was not significantly different among the three groups (*F_2, 17_* = 0.315, *P* = 0.734; Fig. 5d).

### Correlations between RMR and total litter mass

There was a positive relationship between RMR of manipulated lactating voles (n = 23) and total litter mass (r = 0.868, *P*<0.001; Fig. S1).

## Discussion

Our data indicated that reproduction in Brandt's vole is physiologically costly, and was reflected by significantly elevated energy intake and expenditure. However, in contrast to our a priori predictions almost all the immune components that we measured were not suppressed by increased reproductive investment. Specifically, PHA response was not affected, and humoral immunity and innate immunity (i.e. total IgG) were significantly enhanced in lactating voles. In addition, differences in reproductive investment in voles raising both natural and manipulated litters did not affect the immune responses except total IgG. Total IgG was higher in voles with natural large litter size than those with natural small litter size, but decreased in enlarged litter size group compared with control and reduced group; however, these levels were still higher than observed in non-reproductive voles.

### Energetic cost of reproduction

Lactation represents the most energetically demanding periods of the life cycle for small female mammals [5], [37], [38], and energy intake and energy expenditure during lactation are extremely high [5], [39], [40], [41]. A remarkable increase in food intake, RMR and the mass of alimentary tract (see table S1, S2 and S3) assured us that costs of lactation in voles were substantial. Although high energy intake is exported directly to offspring in milk [42], a large proportion is metabolized by females, increasing maternal daily energy expenditure and RMR. The differences observed between lactating and non-lactating animals (e.g. Fig. 1) might actually be the result of some underlying physiological/life-history difference between females which conceived when paired with a male, and those that did not. This is why it is crucial for us to include manipulation of the litter size in Experiment III. Our results showed that RMR increased in lactating voles, especially in the voles with large litter size, and RMR was positively correlated with litter mass (see Fig. S1). These data indicated that the energetic cost of maternal maintenance increased with the increase of energetic investment of lactation. A short-fall in energy intake relative to energy expenditure resulted in declined but non-significant trend of body mass (see Fig. 1a and Fig. S2 and Fig. S3) and mobilization of reserves (body fat) in lactating voles.

Actually, some studies have shown that food intake during peak lactation was limited, and did not increase with the increase of litter size [5], [10], [43], [44]. The apparent physiological limit may be imposed by the capacity of alimentary tract to process food into a form for mobilization [5], [43], and it may underpin an important life-history trait (the maximum litter size) and an important life-history trade-off. It has been suggested that lifetime reproductive success depends more on parental survival than fecundity [45], [46]. Immunity is critical for the survival of small mammals, and it also requires substantial energy [2], [3], [4], [22]. Thus, it is important to note whether the trade-off of resource allocation exists between reproduction and immunity in wild small mammals.

### Effect of reproductive effort on immune function during lactation

Many studies, mostly in birds, have suggested that trade-offs may exist between reproduction and immune function [22], [24], [45], [47], [48], [49], [50], [51]. Many studies of experimentally increased clutch [24], [25] or brood size [48], [52], [53] have detected adverse effect of an increased reproductive effort on different components of the immune system in birds. Increased incubation cost could lead to a lower humoral immune responsiveness and a reduction of lymphocyte levels in eiders *(Somateria mollissima)* [24] and a reduction of survival in great tits *(Parus major)* [54]. Studies which have experimentally increased brood rearing have documented a reduction of T-cell-mediated immunity (measured as a swelling response to PHA) in enlarged broods in pied flycatchers *(Ficedula hypoleuca)* [52] and a suppression of humoral and PHA response in enlarged broods in tree swallow *(Tachycineta bicolor)* [33], [53]. In the current study, however, we found that immune responses were not suppressed in lactating voles and some, for example, KLH-IgG and total IgG were even increased. Moreover, the acquired immune responses did not differ between large litter size group and small litter size groups, both in the experiments where litter size was experimentally manipulated or naturally variable. This result was surprising given that lactating voles, especially for the voles with large litters, exhibited typical characteristics suggesting they were experiencing high energy demands including decreased body mass, fat mass and increased RMR and food intake [14; this study].

Although it is not known why immunity was not suppressed in lactating voles, the vertebrate immune system is very complex. It is likely that different aspects of immunity will respond to energy challenges in different ways [55]. The immune system comprises the acquired arm and the innate arm [56]. Development of the acquired immune system is potentially the largest immunological investment vertebrates make, but the cost of use is modest [4]. Due to this reason, voles might not tend to compromise the acquired arm during lactation which is vital for their long-term survival. Another important component of the immune system is innate immunity (only total IgG measured in the present study). IgG is the most abundant immunoglobulin in circulation, and may represent a state of immunological ‘readiness’ [57]. It is worth noting that total IgG increased in lactating voles, and was also higher in females raising large natural litters. The present result is contrast with that of one previous study in a bird [57]. One possible explanation should be that higher innate immunity may reflect better overall individual condition; thus, those female voles who were in better condition (and had higher IgG levels) could afford to raise larger litters. However, comparing control group (litter size = 6–8) and large group (litter size >8), we found that total IgG was higher in the control group than that in the large group. Thus, the precise mechanism is needed to be clarified in the future study. A potential reason for increased total IgG during lactation may be due to maternal transfer of antibodies which can protect the neonate offspring from infection [58]. In vertebrates, IgG are the primary class of immunoglobulins that transfer via milk [59]. The amount of antibodies transferred to offspring is correlated to the concentration of antibodies in the blood of the females, and mirrors the local disease environment [60], [61]. Therefore, mothers with large litter size had to produce more total IgG due to offspring demand. Only acquired immune responses and total IgG were determined in the present study, and single measures of immunity are probably insufficient to characterize immunocompetence [4], [62].

In addition, Brandt's vole was regarded as an income breeder, which compensates for the energetic demands of reproduction by increasing food intake rather than only mobilizing energy stores [7], [63]. Our data showed that lactating voles dramatically increased their food intake. Overall, maternal food intake increased with increases in litter size and litter mass. The plasticity of digestive tract is necessary for the increased food intake during lactation in several rodents [11], [14], [64]. The digestive tract (including stomach, intestine, caecum and colon) increased in lactating voles, and large litter size group had a greater digestive tract than small litter size group (see table S1, S2, S3). Therefore, the voles may compensate the high energy requirement during lactation by increasing food intake, but not reducing the energy resources allocated to immunity. However, increased feeding rate during lactation might have some other cost, such as increased exposure to predation and/or disease when foraging. Lactating voles exhibited reduced leptin and elevated prolactin, which may be involved in the regulation of food intake and energy expenditure [14], [15], [65], [66]. Moreover, these two hormones may be important regulators of the reproductive and immune systems and their interactions [4], [49], [67]. However, we did not find any correlations between these two hormones and immunity in this study. ‘Stress hormone’ corticosterone has been used by ecologists as an indicator of physiological stress in wild vertebrates [68], and it seems that lactation (no change in corticosterone) is not a kind of stress for voles. Although we do not detect any correlations between the hormones and immune function in voles in the present study, fluctuations in these hormones must be helpful for maintaining normal immune function during lactation. Thus, more studies should be conducted to illustrate the roles of these hormones in mediating immune activity in wild animals with a seasonal breeding cycle.

Another possible contributing factor is that Brandt's voles may have experienced great exposure to pathogens and parasites in the wild, especially during the reproductive season [69], thus they may have evolved a strategy to increase investment in immune function at the cost of resources available for their offspring (e.g. reduced growth rate of the large litter size group) [70]. Given the changes in immunity can have marked consequences for disease resistance and long-term survival costs [48], [71], animals with high exposure to pathogens and parasites may be unwilling to down regulate immune function.

In conclusion, our results suggest that immunity is not suppressed in Brandt's voles during lactation even when reproductive investment was experimentally increased. Interestingly, innate immunity was even enhanced during lactation. Although there might be a negligible trade-off between litter size and innate immunity in terms of not enhanced serum total IgG in the experimental enlarged group, overall, these data do not support the idea that a trade-off exists between reproduction and immunity in lactating voles. Taken together, immune function is unlikely to be an important aspect mediating the trade-off between reproduction and survival in lactating Brandt's voles.

## Materials and Methods

### Ethics statement

All animal procedures were reviewed and approved by the Institutional Animal Care and Use Committee of the Institute of Zoology, Chinese Academy of Sciences (Permit Number: IOZ11012). All researchers and students had been certified before performing animal studies.

### Study species

Brandt's voles inhabit mainly the grasslands of Inner Mongolia of China, Mongolia, and the Baikal region of Russia. They were non-hibernating herbivores and polygamy. They hoard food in the late fall and living in groups during cold winter. In the wild 90% of female voles commenced breeding in April, and the reproductive season lasted till August. During this period, one female could raise 1–2 litters, the length of gestation was 21 days, and the litter size ranged from 2 to 13. In the wild, Brandt's vole's life span is around 14 months, and in the laboratory voles can live for 31 months [69]. Previous studies have shown that energy intake and resting metabolic rate increased, and body fat was depleted during lactation [14].

### Animals and housing conditions

One hundred and eighteen virgin adult female Brandt's voles, weighing 40–55 g and aged 120–150 days old, were used in this study. They were the offspring of voles from our laboratory colony. Voles were kept individually in plastic cages (30 cm×15 cm×20 cm) under a 16 h: 8 h light/dark cycle and room temperature (21±1°C). Commercial standard rabbit pellets (Beijing KeAo Feed Co., Beijing, China) and water were provided *ad libitum*. All the females were randomly paired with males for 1 day and then were immediately separated from the males. The day of parturition was designated as day 0 of lactation [42]. To assess humoral and cellular immunity during lactation, each group was divided into two groups which were immunochallenged with KLH (Sigma 7017) and phytohemagglutinin (PHA; PHA-P, Sigma L-8754) solution: KLH group and PHA group. All animals were naïve to KLH and PHA. Animals in KLH group and PHA group were sacrificed on day 18 and day 15 of lactation which are late peak lactation. Each vole was euthanized by CO_2_ asphyxiation between 0900 and 1100 h, and trunk blood was collected which were allowed to clot for 30 min at 4°C and centrifuged at 4°C for 30 min at 3000 r. p. m. Sera were collected and stored in sealable polypropylene micro-centrifuge tubes at −80°C until assay for total IgG, anti-KLH IgG, leptin, prolactin and corticosterone.

### Experiment I

In the first experiment we examined whether immune function was compromised in lactating voles compared with non-reproductive voles. Voles whose litter size was 6–8 were defined as the lactating group (L_0_, n = 13). Animals that were not pregnant or lactating were defined as the non-reproductive group (N, n = 14). Each group was divided into two further groups depending on the immune function assays performed: the KLH group (L_0_K and NK, n = 7 and n = 7, respectively) and the PHA group (L_0_P and NP, n = 6 and n = 7, respectively).

### Experiment II

The second experiment explored the effect of natural large and small litter sizes, which were presumed to reflect different reproductive effort, on immune function in lactating voles. Lactating females whose litter size was more than 8 or less than 6 were selected and defined as the large group (L, n = 13) or the small group (S, n = 24), respectively. Each group was divided into two further groups as in experiment I: the KLH group (LK and SK, n = 6 and n = 14, respectively) and the PHA group (LP and SP, n = 7 and n = 10, respectively).

### Experiment III

To further test the relationship between reproductive effort and immune function, in the final experiment we manipulated litter size to examine the effect of increased or decreased reproductive effort on the immune function. Animals whose original litter size at birth was 6–8 were used in this experiment. We manipulated litter size by adding or removing pups on the day of parturition. Litters with same parturition date were mixed together and assigned randomly to females. By adding or removing 5 pups, we assigned pups randomly to three treatment groups: E, enlarged group (initial litter size 6–8, with 5 pups added); C, control group (with the initial litter size unchanged); R, reduced group (initial litter size 6–8, with 5 pups removed). Maternal voles easily accept foreign pups, as the survival of offspring did not differ between original pups and cross-fostered pups. Each group was divided into two groups, KLH group (EK, CK and RK, n = 7, n = 6 and n = 7, respectively) and PHA group (EP, CP and RP, n = 7, n = 6 and n = 8, respectively).

### Body mass, food intake and reproductive performance

Body mass and food intake were measured at 9:00–11:00 every three days for 9 days before mating and throughout lactation. Dry matter intake was calculated from the difference between the dry food given and the dry food residue [food given× (1-water content)−food residue× (1-water content)]. Food samples were taken to determine the water content (5.6±0.7%, *N* = 15). Litter size and litter mass were also recorded every three days during lactation.

### Resting metabolic rate measurements

On day 13 of lactation, RMR of voles from the PHA groups of the three experiments was measured by using an open-flow respirometry system (Sable, FoxBox, USA) at 30±0.5°C (within thermal neutral zone 27.5–32.5°C of Brandt's vole [72], which was controlled with a incubator (Yiheng Model LRH-250, Shanghai, China) as described previously [73]. Further details can be found in the Appendix S1 in supporting information.

### Immune response measurements

#### Serum total IgG assay

Serum samples from PHA groups of the three experiments were used to determine serum total IgG. Total IgG is one component of innate immunity which may be particularly important for survival in the wild [57].The concentration of total IgG was measured by rat IgG ELISA (enzyme-linked immunosorbent assay) kit (RapidBio Lab, Calabasas, CA, USA). The sensitivity by this assay is 1.0 µg/ml when using 10 µl serum samples. Inter- and intra-assay variations were both <15%.

#### Humoral immunity

To assess humoral immunity, animals received a single subcutaneous injection of 100 µg of KLH suspended in 0.1 ml sterile saline on day 8 of peak lactation. KLH is a specific non-replicating antigen which induces a robust antibody response without inducing fever or making the animal sick [74]. Animals in all groups were lightly anesthetized with isoflurane (Shandong LiNuo Pharmaceutical) and bled from retro-orbital sinus 5 days post injection to measure anti-KLH IgM concentrations between 0900 and 1100 h.

IgM is the major class of antibody early in a primary antibody response, and IgG is the predominant immunoglobulin class present in the blood following an immune challenge [75]. Enzyme-linked immune-sorbent assay (ELISA) was used to measure serum anti-KLH IgM and IgG concentrations according to [36], [75]. The Appendix S1 has displayed further details.

#### PHA response

To measure delayed-type hypersensitivity responses which are localized antigen-specific responses eliciting swelling and redness at the site of antigen injection in immunized animals, we injected subcutaneously 0.1 mg of PHA (PHA-P, Sigma L-8754) dissolved in 0.03 ml of sterile PBS (pH 7.4) in the middle of the left footpad of PHA group around 0900. Prior to injection, the footpad thickness of left hind foot was measured to the nearest 0.01 mm with a micrometer (Tesa Shopcal, Swiss). Six hours after injection, we measured footpad thickness at the injection site. The PHA response was calculated as the difference between pre- and post-injection measurements divided by initial footpad thickness (PHA response = (post PHA−pre PHA)/pre PHA). Each measurement of PHA response was replicated six times [76], [77]. The pre-experiment showed that the maximum PHA response occurs after 6 h of PHA injection (unpublished data).

The methods for measuring body composition, organ mass, blood glucose and serum hormones can be found in Appendix S1.

### Statistical analysis

Data were analyzed using SPSS 17.0 software (SPSS Inc., Chicago, IL, USA). Prior to all statistical analyses, data were examined for normality of variance using the Kolmogorov-Smirnov test. Differences in body mass, litter mass and mean pup mass were analyzed by one-way repeated-measures ANOVA, while differences in food intake were analyzed by one-way repeated-measures ANCOVA with body mass as covariate followed by Tukey's honestly significant difference post hoc comparisons. Differences in RMR, body compositions and organ mass were analyzed by one-way ANCOVA with body mass as covariate followed by Tukey's honestly significant difference post hoc comparisons. Serum hormones (leptin, corticosterone and prolactin), immune responses (serum anti-KLH IgM, serum anti-KLH IgG, PHA response) were analyzed by independent-samples T test in the first and second experiment. Group differences in serum hormones and immune responses were analyzed by one-way ANOVA in the third experiment. Finally, Pearson correlation analysis was performed to determine the correlations between RMR and total litter mass. Differences between group means were considered statistically significant at P<0.05. All the variables analyzed (except humoral immunity) are from the PHA group of the three experiments.

## Supporting Information

Appendix S1 **Supplementary Appendix of methods, including resting metabolic rate, measuring serum anti-KLH IgM and IgG concentrations, measuring body composition and organ mass and serum hormones.**
(DOC)

Figure S1 **Correlations between RMR and total litter mass.**
(TIF)

Figure S2 **Changes of maternal body mass in large and small litter size group before and after lactation.** L represents large litter size group, and S represents small litter size group.
(TIF)

Figure S3 **Changes of maternal body mass in manipulation experiment before and after lactation.** E represents enlarged litter size group, C represents non-manipulated litter size group, and R represents reduced litter size group.
(TIF)

Table S1 **The effects of lactation on body composition, wet organ mass in female Brandt's voles.**
(DOC)

Table S2 **The effects of litter size on body composition, wet organ mass and hormones in Brandt's voles.**
(DOC)

Table S3 **The effects of manipulation on body composition, wet organ mass and hormones in Brandt's voles.**
(DOC)
