# Supplementary material for: No Evidence for a Trade-Off between Reproductive Investment and Immunity in a Rodent
Source: PLoS One. 2012 May 23;7(5):e37182. doi: 10.1371/journal.pone.0037182 (PMC3359356; doi:10.1371/journal.pone.0037182)
Supplement: Appendix S1 — Supplementary Appendix of methods, including resting metabolic rate, measuring serum anti-KLH IgM and IgG concentrations, measuring body composition and organ mass and serum hormones. (DOC) [file pone.0037182.s001.doc]

Supplementary Appendix

**Supplementary method of resting metabolic rate**

Briefly, the voles were placed in a transparent plastic chamber (volume 1.4L, 20.5cm×13.4cm×8.4cm) with small pieces of paper to absorb animal wastes. Both the incurrent and excurrent air was dried using a column filled with DRIERITE desiccants (W.A. Hammond Drierite Co. Ltd, USA). Before the air flow into the chamber, it was warmed firstly through a copper tube. The flow rate of air passing the chamber was 400–600 ml min^−1^; the subsample rate was approximately 100 ml min^−1^. The gas was submitted to oxygen analyzer for analysis (Sable Foxbox). The baseline of oxygen and carbon dioxide concentration was measured at the beginning and end of each metabolic measurement, respectively. Oxygen consumption of each animal was recorded for 2h at intervals of 10s. The average of 5-minute continuous stable minimum recordings was taken to calculate RMR. Before and after measurement, body mass (Sartorius, to ± 0.1 g) and rectal body temperature (TES-1310, to ± 0.1 °C) were monitored immediately .

**Supplementary method of measuring serum anti-KLH IgM and IgG concentrations**

Micro-titer plates were coated with 100 ml 0.5 mg ml^-1^ KLH in sodium bicarbonate buffer (pH 9.6) overnight at 4^o^C and washed with phosphate buffered saline (PBS) containing 0.05% Tween 20 (PBS-T, pH 7.4) three times, then blocked with 5% nonfat dry milk in PBS-T overnight at 4^o^C to reduce nonspecific binding, and washed again with PBS-T three times. Thawed serum samples were diluted 1: 20 with PBS-T, and 150 μl of each serum dilution was added in duplicate to the wells of the antigen-coated plates. Positive-control samples (pooled sera from repeatedly KLH-challenged voles determined to have high levels of anti-KLH IgM and IgG, similarly diluted with PBS-T) and negative control samples (pooled sera from KLH naive voles, similarly diluted with PBS-T) were added in duplicate. Plates were sealed and incubated at 37^o^C℃ for 3 h, then washed with PBS-T three times. Secondary antibody (alkaline phosphatase-conjugated anti-mouse IgG diluted 1: 2,000 with PBS-T; alkaline phosphatase-conjugated anti-mouse IgM diluted 1: 500 with PBS-T, Sigma Chemical, St. Louis, MO) was added to the wells, and the plates were sealed and incubated for 1 h at 37^o^C. Plates were washed again with PBS-T, and 150μl enzyme-substrate p-nitrophenyl phosphate (Sigma chemical, St Louis, MO; 1mg ml^-1^ in diethanolamine substrate buffer) was added to each well. Plates were protected from light during the enzyme-substrate reaction, which were terminated after 20 min by adding 50 μl of 1.5 mol L^-1^ NaOH solution to each well. The optical density (OD) of each well was determined using a plate reader (VersaMax™) equipped with a 405-nm-wavelength filter and the mean OD for each set of duplicate wells was calculated. To minimize intra-assay variability, the mean OD for each sample will be expressed as a percentage of its plate-positive-control OD for statistical analysis .

**Supplementary method of body composition and organ mass**

At the end of the experiment all voles were sacrificed between 0900 and 1200, gastrointestinal tract (stomach, small intestine, colon, and cecum) were extracted firstly, weighed with and without content (Mettler PB153, to 1 mg), respectively. Finally, the visceral organs, including heart, liver, lung, spleen, kidneys and thymus were removed and weighted respectively. All organs and carcass were dried to constant mass in an oven at 60 °C and reweighed to get the dry mass. Total body fat mass was extracted from the dried carcass by petroleum ether extraction in a Soxhlet apparatus (Foss Model Soxtec Avanti 2050).

**Supplementary method of serum hormones**

Serum prolactin hormones were determined by radio-immunoassay using RIA kits from Beijing Institute of Northern Biotech. Inter- and intra-assay variations were 15% and 10%, respectively.

Serum leptin concentrations were assayed by radio-immunoassay (RIA) with a ^125^I multi-species kit (Millipore Corporation, Cat. No. XL-85 K, MO, USA). The range of the assay kit was 1 and 50 ng/ml. The inter- and intra-assay coefficients of variation were <8.7% and<3.6%, respectively.

Serum corticosterone levels were assayed by rat corticosterone ELISA (enzyme-linked immunosorbent assay) kit (Cat. No. HR083, RapidBio Lab. Calabasas, CA, USA). The lowest concentration of corticosterone that could be detected by this assay was 0.7nmol/L when using 25μl serum sample. Inter- and intra-assay variations were <7% and < 5%, respectively (Xu & Wang 2010).

**Reference**

Chi, Q. S. & Wang, D. H. (2010) Thermal physiology and energetics in male desert hamsters *(Phodopus roborovskii)* during cold acclimation. *Journal of Comparative Physiology B-Biochemical Systemic and Environmental Physiology,* **181**, 91-103.

Demas, G. E., Drazen, D. L. & Nelson, R. J. (2003) Reductions in total body fat decrease humoral immunity. *Proceedings of the Royal Society Series B-Biological Sciences,* **270**, 905-911.

Xu, D. L. & Wang, D. H. (2010) Fasting suppresses T cell-mediated immunity in female Mongolian gerbils (*Meriones unguiculatus*). *Comparative Biochemistry and Physiology Part A-Molecular & Integrative Physiology,* **155**, 25-33.

Zysling, D. A. & Demas, G. E. (2007) Metabolic stress suppresses humoral immune function in long-day, but not short-day, Siberian hamsters (*Phodopus sungorus*). *Journal of Comparative Physiology B-Biochemical Systemic and Environmental Physiology,* **177**, 339-347.
