# Supplementary material for: No Evidence for a Trade-Off between Reproductive Investment and Immunity in a Rodent
Source: PLoS One. 2012 May 23;7(5):e37182. doi: 10.1371/journal.pone.0037182 (PMC3359356; doi:10.1371/journal.pone.0037182)
Supplement: Table S1 — The effects of lactation on body composition, wet organ mass in female Brandt's voles. (DOC) [file pone.0037182.s005.doc]

Table S1. Effect of lactation on body composition, wet organ mass and blood glucose in female Brandt’s voles

| Parameters | N | L_0_ | P |
| --- | --- | --- | --- |
| Body fat mass(g)  Body fat content(body fat mass/wet carcass mass) | 18.654±1.654^a^  39.929±3.031^a^ | 6.52±1.805^b^  17.97±3.3.09^b^ | P<0.01  P<0.01 |
| Thymus(mg) | 10.285±1.724 ^a^ | 5.166±1.862 ^b^ | P<0.05 |
| Spleen(mg) | 35.714±5.870 | 43.667±5.696 | ns |
| Heart(g) | 0.230±0.015 | 0.254±0.014 | ns |
| Liver(g) | 1.803±0.127 ^a^ | 2.713±0.115 ^b^ | P<0.05 |
| Lungs(g) | 0.307±0.043 | 0.346±0.039 | ns |
| Kidneys(g) | 0.440±0.040 | 0.569±0.037 | ns |
| Stomach with content(g) | 1.104±0.221 ^a^ | 1.838±0.199 ^b^ | P<0.05 |
| Stomach(g) | 0.317±0.029 ^a^ | 0.434±0.026 ^b^ | P<0.05 |
| Small intestine with content(g) | 1.446±0.148 ^a^ | 2.780±0.134 ^b^ | P<0.05 |
| Small intestine(g) | 0.649±0.096 ^a^ | 1.010±0.087 ^b^ | P<0.05 |
| Caecum with content(g) | 2.682±0.633 | 5.409±0.571 | ns |
| Caecum(g) | 0.454±0.086 ^a^ | 0.699±0.078 ^b^ | P<0.05 |
| Colon with content(g) | 0.660±0.218 ^a^ | 1.386±0.197 ^b^ | P<0.05 |
| Colon(g) | 0.293±0.044 ^a^ | 0.506±0.039 ^b^ | P<0.05 |

Values are means ± s.e.m. significant differences are indicated by different superscripts in each row if P < 0.05.
