# Supplementary material for: No Evidence for a Trade-Off between Reproductive Investment and Immunity in a Rodent
Source: PLoS One. 2012 May 23;7(5):e37182. doi: 10.1371/journal.pone.0037182 (PMC3359356; doi:10.1371/journal.pone.0037182)
Supplement: Table S2 — The effects of litter size on body composition, wet organ mass and hormones in Brandt's voles. (DOC) [file pone.0037182.s006.doc]

Table S2. The effects of litter size on body composition, wet organ mass, blood glucose and hormones in Brandt’s voles.

| Parameters | L | S | P |
| --- | --- | --- | --- |
| Body fat mass(g)  Body fat content(body fat mass/wet carcass mass) | 6.873±0.936  16.507±2.169 | 7.844±0.712  19.416±1.649 | ns  ns |
| Thymus(mg) | 6.285±1.232 | 5.4±1.081 | ns |
| Spleen(mg) | 48.428±4.979 | 40.800±4.369 | ns |
| Heart(g) | 0.259±0.015 | 0.230±0.009 | ns |
| Liver(g) | 2.845±0.24 | 2.239±0.105 | P<0.05 |
| Lungs(g) | 0.298±0.019 | 0.293±0.023 | ns |
| Kidneys(g) | 0.568±0.044 | 0.477±0.015 | P<0.01 |
| Stomach with content(g) | 2.021±0.445 | 1.433±0.133 | ns |
| Stomach(g) | 0.432±0.031 | 0.379±0.016 | ns |
| Small intestine with content(g) | 3.318±0.266 | 2.474±0.815 | ns |
| Small intestine(g) | 1.033±0.111 | 0.815±0.041 | ns |
| Caecum with content(g) | 4.722±0.34 | 4.348±0.337 | P<0.05 |
| Caecum(g) | 0.754±0.056 | 0.696±0.078 | ns |
| Colon with content(g) | 1.415±0.164 | 1.268±0.188 | ns |
| Colon(g) | 0.566±0.053 | 0.483±0.037 | ns |
| Leptin(ng/ml) | 3.307±0.146 | 2.986±0.148 | ns |
| Prolactin(ng/ml) | 187.087±9.718 | 163.685±15.957 | ns |
| Corticosterone(nmol/l) | 3.215±0.118 | 2.891±0.189 | ns |

Values are means ± s.e.m. significant differences are indicated by different superscripts in each row if P < 0.05.
