# Supplementary material for: No Evidence for a Trade-Off between Reproductive Investment and Immunity in a Rodent
Source: PLoS One. 2012 May 23;7(5):e37182. doi: 10.1371/journal.pone.0037182 (PMC3359356; doi:10.1371/journal.pone.0037182)
Supplement: Table S3 — The effects of manipulation on body composition, wet organ mass and hormones in Brandt's voles. (DOC) [file pone.0037182.s007.doc]

Table S3. The effects of manipulation on body composition, wet organ mass, blood glucose and hormones in Brandt’s voles.

| Parameters | E | C | R | P |
| --- | --- | --- | --- | --- |
| Body fat mass(g)  Body fat content(body fat mass/wet carcass mass) ％ | 10.154±1.842  23.767±3.978 | 8.761±2.122  20.648±4.582 | 8.46±1.957  21.171±4.226 | ns  ns |
| Thymus(mg)  Spleen(mg) | 4.429±1.017  36.285±6.234 | 5.333±1.119  43.667±6.623 | 5±1.166  39.125±7.182 | ns  ns |
| Heart(g) | 0.235±0.010 | 0.254±0.011 | 0.206±0.011 | ns |
| Liver(g) | 2.756±0.125 | 2.713±0.138 | 2.114±0.144 | ns |
| Lungs(g) | 0.323±0.029 | 0.346±0.031 | 0.265±0.033 | ns |
| Kidneys(g) | 0.513±0.024 | 0.569±0.027 | 0.430±0.028 | ns |
| Stomach with content(g) | 1.358±0.123 ^a^ | 1.838±0.135^b^ | 0.937±0.141^a^ | P<0.05 |
| Stomach(g) | 0.344±0.020 ^a^ | 0.434±0.022 ^b^ | 0.303±0.023 ^a^ | P<0.05 |
| Small intestine with content(g) | 2.791±0.202 | 2.780±0.222 | 2.212±0.232 | ns |
| Small intestine(g) | 0.976±0.116 | 1.010±0.128 | 0.699±0.133 | ns |
| Caecum with content(g) | 4.730±0.539 | 5.409±0.593 | 4.118±0.618 | ns |
| Caecum(g) | 0.546±0.055 | 0.699±0.061 | 0.479±0.063 | ns |
| Colon with content(g) | 1.339±0.154 | 1.386±0.170 | 1.132±0.177 | ns |
| Colon(g) | 0.480±0.065 | 0.506±0.072 | 0.448±0.075 | ns |
| Leptin(ng/ml) | 2.515±0.202 | 2.717±0.217 | 3.178±0.258 | ns |
| Prolactin(ng/ml) | 202.432±15.794 | 176.673±16.305 | 169.69±22.245 | ns |
| Corticosterone(nmol/l) | 3.751±0.325 | 3.801±0.339 | 3.334±0.209 | ns |

Values are means ± s.e.m. significant differences are indicated by different superscripts in each row if P < 0.05.
